# Medical History from the Earliest Times

**Published:** 1893-02-25

**Authors:** E. T. Withington


### Medical History from the Earliest Times.

By E. T. Withington, M.A., M.B. Oxon.

### XLIV.—UNPROFESSIONAL MEDICINE IN THE MIDDLE AGES.

In mediæval medical writings we frequently come across the terms "laicus" and "idiota," applied to practitioners who had received no regular education, *i.e.*, were not "clerks," and who were acquainted only with their native tongue. The Greek "idiot" was a man who took no part in State affairs, the mediæval "idiot" was one who knew no Latin. But the words are also used to denote the amateurs of the healing art, who were as numerous then as now, and it may be interesting to consider some aspects of this unprofessional medicine as exemplified by Kings, saints, bishops, priests, and the general public.

The history of "touching" for scrofula by English sovereigns has been so often related that we may confine ourselves to a short account of the practice by French Kings. In the opinion of St. Thomas Aquinas there is no better proof that divine grace is given to anointed monarchs, than the undoubted fact that the power of healing diseases was thereby bestowed on that strange "eldest son of the Church," Clovis the Frank, A.D. 496. But he does not mention scrofula, and the earliest notice of a special power of healing that disease, is probably the following passage from Abbot Guibert de Nogent, author of the famous "Gesta Dei per Francos," "What a prodigy is that which we see practised by our sovereign Lord Louis [the Fat, 1100—1137]. I have seen those who have swellings on the neck or elsewhere run in crowds to be touched by him, while I was present and trying to keep them off. But he, with his natural kindness, gently beckoned them to him, and humbly signed them with the sign of the Cross. His father Philip often performed this glorious miracle, but lost the power through falling into some sin. I pass over what other Kings do in this way, but I know the King of England never attempts it." Henry IV. is said to have healed more than 1,500 patients, and to have touched nearly four times as many every year. "After the ceremony," says [an ancient chronicle," the King wiped his fingers on a cloth dipped in wine and water, and then dined, though he seldom had much appetite thereto, because of the unpleasant smells and sights; but charity overcometh all things." The custom was continued till the Revolution, and when, about 1750, D'Argenson sent the Ministers of Louis XV. details of a case which had actually recovered after being "touched," he received the crushing reply: "Monsieur, the prerogative of the Kings of France to cure scrofula is proved by such unquestionable evidence that it is unnecessary to confirm it by particular instances." Many cases of failure, however, occurred, even in the ages of faith, one of which is of special interest. In 1483 the crafty and superstitious Louis XI. sent for St. Francis of Paul, who had wrought many wonders in Italy, to heal him of the results of an apoplexy. That holy man suffered from scrofula, but the historian is obliged to confess that neither the saint could cure the King nor the King the saint.

The power of mediæval saints to heal the sick, like that of the Kings of France, is so notorious that it is needless to give examples; but one of their number has special claims to a place in the history of medicine, St. Hildegard of Bingen (1098—1179), who shares with her contemporary, Avenzoar, the honour of having first mentioned the itch mite. According to some modern writers she established a school of nurses (!), but there is no mention of this in her letters or authentic biographies. Her own patients, at any rate, required no nursing, for she cured them in the miraculous manner appropriate to her saintly character, and we are told that hardly any sick persons applied to her who were not instantly healed. But for the benefit of those less highly favoured, St. Hildegard wrote several medical books, of which the best known is the "Physica," a description of the nature and medical properties of minerals, herbs, fishes, birds, and animals. We shall again refer to this work when discussing the doctrine of "Signatures," but the reader may be interested by the following description of the unicorn, in which it is hard to say whether the saint is consciously or unconsciously humorous. "As the serpent in the Garden of Eden avoided the man and gazed at the woman, so this animal flees from men and follows females. A certain philosopher, skilled in the ways of beasts, had long hunted a unicorn, but could not catch him, whereat he marvelled greatly. But one day he went hunting with a company of men and women, and the unicorn seeing the girls, slackened his pace, sat on his hind legs, and stared at them. And the philosopher, when he had diligently considered this, saw that the animal might thus be caught, so he came up behind him and captured him. For the unicorn, when he sees a girl, marvels that she has no beard, and yet has the form of man, and if there are several girls he marvels the more, and is caught the more easily. Get a unicorn's liver and make it into an ointment with yolk of egg. There is no leprosy of any kind which this will not cure, if the patient uses it often, unless his death is fore-ordained, or God willeth not that he be healed. Make a belt of unicorn's skin, and wear it next your own, and no pestilence or fever will harm you."

The monasteries, as we have seen, were the mediæval dispensaries, and the clergy were not only often regular members of the medical profession, but held that the treatment of the numerous so-called supernatural diseases belonged especially to their order. In times of pestilence bishops wrote pastoral letters exhorting their flocks to avoid bodily and cleave to spiritual aids, for it is better to fall into the hands of God than into the hands of man. Plagues are divine punishments, and the temptation to use physical means of cure must be avoided as a snare and a sin, "lest haply they be found even to fight against God." It was perhaps well that they abstained from recommending medicines, if we may judge from the following example given by Guy of Chauliac. Speaking of opiates, he observes that in administering such drugs the dose and time must be carefully considered. "It was this that made the physicians suspect those lozenges which the Lord Bishop of Riegs recommended to the Lord Bishop of Marseilles, who suffered from a painful strangury, after taking whereof he died in his sleep."

He then gives the prescription, which included five drachms of opium, and one of henbane! As an agreeable contrast to the above, it is interesting to find that in 1255 Walter de Kirkham, Bishop of Durham, ordered all the clergy of his diocese to solemnly exhort mothers from the pulpit not to take their babies to bed with them, for through this habit many were suffocated, an excellent piece of episcopal advice which might be repeated with advantage.

In modern times the clergyman and the Lady Bountiful are the most prominent of amateur practitioners, and we may conclude with two mediæval examples of these forms of unprofessional medicine. The following is the 73rd "Observation" of Antony Benivieni (1440-1502), a physician whom we shall meet again shortly: "An acquaintance of mine, Michelotti, was troubled with hernia, and went to a priest who professed to cure that disorder. Without reducing the rupture, the latter immediately put on a tight bandage. The bowel remained so compressed for a week, and became inflamed, causing the patient great pain. Suddenly the pain ceased, and he became delirious, so, at last, I was sent for. Having felt his pulse, which was hardly perceptible, and seen his pale face and sunken eyes, I ordered the priest to be summoned. Then I removed the bandage, and saw the whole part blackened, with an ulcer showing the putrid intestine. He died in a few hours, leaving us a useful example and warning not to entrust our sick bodies to priests, who are only required to cure the soul, but to the more learned physicians."

Sometimes, however, even learned physicians and surgeons quote lay prescriptions with approval. Thus Lanfranc, after describing how to make a truss with a piece of sheet iron and a bandage, adds: "Give the patient also every day five drachms of powdered valerian root in wine. This powder has a marvellous effect, as I have often proved. I got the receipt from one who had it of a certain noble lady, who treated all ruptures therewith."

